# A Systematic Review of Bacterial Sampling Collection for Veterinary Microbiology in Companion Animals

**DOI:** 10.3390/vetsci13020126

**Published:** 2026-01-28

**Authors:** Inês C. Rodrigues, Joana C. Prata, Ângela Pista, Paulo Martins da Costa

**Affiliations:** 1 School of Medicine and Biomedical Sciences, University of Porto (ICBAS-UP), Rua de Jorge Viterbo Ferreira, 228, 4050-313 Porto, Portugal; icrodrigues@icbas.up.pt; 2 Interdisciplinary Centre of Marine and Environmental Research (CIIMAR), Terminal de Cruzeiros do Porto, de Leixões, Avenue General Norton de Matos s/n, 4450-208 Matosinhos, Portugal; 3 i4HB—Institute for Health and Bioeconomy, University Institute of Health Sciences (CESPU), 4585-116 Gandra, Portugal; joana.prata@iucs.cespu.pt; 4 UCIBIO—Applied Molecular Biosciences Unit, Translational Toxicology Research Laboratory, University Institute of Health Sciences (1H-TOXRUN, IUCS-CESPU), 4585-116 Gandra, Portugal; 5 National Reference Laboratory for Gastrointestinal Infections, Department of Infectious Diseases, National Institute of Health Doutor Ricardo Jorge, Avenue Padre Cruz, 1649-016 Lisbon, Portugal; angela.pista@insa.min-saude.pt

**Keywords:** companion animals, microbiological sampling, bacterial culture, sample handling and transport

## Abstract

Accurate microbiological diagnosis in companion animals relies heavily on appropriate sample collection, handling, storage, and transport. This systematic review, conducted according to the PRISMA 2020 guidelines, aimed to assess current microbiological sampling procedures used in dogs and cats, from specimen collection to laboratory submission. Seventeen studies published in English over the past decade met the inclusion criteria. Considerable variability in sampling practices was observed across different anatomical systems. Although swabs were the most frequently employed sampling method, their use is limited by reduced representativeness and an increased risk of contamination, whereas tissue biopsies and aspirates provide more reliable diagnostic results. Sampling practices were generally more consistent for urine samples; however, pre-laboratory factors, such as prior antimicrobial exposure, as well as transport and storage conditions, were inconsistently reported, hindering reproducibility and standardisation. The anatomical site sampled and the suspected pathogen determined whether aerobic or combined aerobic–anaerobic cultures were recommended. Overall, the findings reveal considerable methodological gaps and underscore the need for harmonised, evidence-based guidelines to support accurate and clinically relevant bacterial culture in veterinary diagnostics. Standardised protocols, developed collaboratively by clinicians and diagnostic laboratories, have the potential to improve diagnostic consistency, strengthen antimicrobial stewardship, enhance surveillance, and ultimately benefit clinical outcomes within One Health initiatives.

## Simple Summary

Accurate diagnosis of bacterial infections in dogs and cats depends on proper collection, handling, storage, and transport of samples. This review examined current practices for taking and processing samples for bacterial testing, based on seventeen studies published over the last ten years. The results showed that sampling methods vary widely across body sites. Swabs were most commonly used, but tissue and fluid samples generally provide more reliable results. Key information, such as prior antibiotic use and storage or transport conditions, was often missing, which can affect test accuracy. The choice of culture method and storage depends on the suspected bacteria and the body site sampled. Overall, there are significant gaps in standardisation. Developing clear, evidence-based guidelines could improve test reliability, support responsible antibiotic use, and ultimately enhance the health of companion animals and the wider community.

## 1. Introduction

Microbiological diagnosis is a cornerstone of veterinary clinical practice, underpinning the identification of infectious agents and guiding antimicrobial therapy [1,2]. Bacterial infections are common in companion animals and often require prompt and accurate diagnosis to enable effective treatment [3]. However, the increasing prevalence of antimicrobial resistance (AMR) among veterinary pathogens poses a major clinical and public health challenge [3,4]. In this context, the need for accurate microbiological diagnosis becomes even more critical. Laboratory-guided antimicrobial therapy is crucial not only for improving clinical outcomes but also for promoting responsible antimicrobial use [5].

One important, though often underestimated, factor influencing diagnostic reliability is the quality of sample collection [6]. These methods, which include the technique used, the timing of collection and the conditions of transport and storage, determine the accuracy of culture and subsequent bacterial identification [6]. Equally critical is the quality and completeness of the clinical information accompanying the sample, as this contextual data is essential for the correct interpretation of microbiological findings and for distinguishing pathogens from contaminants or commensal organisms [6]. The diagnostic value of microbiological testing may be compromised by inadequate or improperly collected samples, as well as by insufficient or misleading clinical information, potentially leading to contamination, false-negative results, failure to recover the causative pathogen, or the isolation and misinterpretation of non-pathogenic or commensal microorganisms [5,7].

Despite its recognized importance, the use of these diagnostic tools, particularly culture and antimicrobial susceptibility testing, remains underused in veterinary settings. Previous studies conducted among European veterinarians have highlighted that faster results and reduced costs are key factors that could increase the use of antimicrobial susceptibility testing in this field [2,8]. Nonetheless, additional barriers, such as a lack of confidence in microbiological results, reliance on clinical assumptions and limited understanding of how to interpret susceptibility data, continue to hinder wider adoption [8]. Furthermore, antimicrobial susceptibility methods are not yet fully standardized, and for several antimicrobial agents and bacterial species, clinical breakpoints are lacking or incomplete, with epidemiological cut-off values often being the only available interpretative criteria, further complicating diagnostic reliability [9].

Sample collection practices in veterinary microbiology also remain highly variable, with limited evidence-based guidelines tailored specifically to small animal species [10]. This variability compromises diagnostic accuracy and may hinder appropriate antimicrobial selection, indirectly contributing to the AMR crisis.

While resources describing the handling of veterinary microbiological specimens upon their arrival at diagnostic laboratories are available, guidance for practitioners on best practices for sample collection remains scarce [10]. To date, no systematic review has consolidated the available evidence on microbiological sampling methods in dogs and cats. A comprehensive synthesis of current practices is therefore necessary to identify gaps, promote harmonisation, and strengthen the integration of microbiological data into both clinical decision-making and AMR surveillance.

The present systematic review aims to identify, summarise and evaluate the current sampling procedures used for microbiological diagnosis in companion animal veterinary practice, from sample collection to submission to the microbiology laboratory. Focusing on dogs and cats, the review examines collection techniques, specimen handling, and transport conditions that influence the reliability of microbiological results. The overarching goal is to support veterinary clinicians and diagnostic laboratories in improving sampling quality, thereby enhancing diagnostic accuracy, advancing antimicrobial stewardship, and ultimately safeguarding both animal and public health.

## 2. Materials and Methods

This systematic review was performed according to PRISMA 2020 (Preferred Reporting Items for Systematic Reviews and Meta-analyses) guidelines [11], with the protocol preregistered on OSF (timestamped 12 January 2026; registration number 10.17605/OSF.IO/BSVNJ).

### 2.1. Selection Strategy

In April 2025, an independent researcher (I.C.R.) conducted a systematic search of the PubMed and ScienceDirect databases, covering the last ten years (from 2015 onwards). This timeframe was chosen to capture the most recent and relevant evidence available in the field, in line with current recommendations for evidence-based reviews [12]. The aim was to identify peer-reviewed studies reporting sampling methods for microbiological diagnosis in dogs and cats, within a veterinary clinical context.

The following search terms were used in combination: (“microbiological diagnosis” OR “microbiological testing” OR “bacterial identification”) AND (“sampling method” OR “sample collection”) AND (“veterinary” OR “dog” OR “cat”).

Titles and abstracts were screened to assess eligibility for inclusion. Only peer-reviewed studies were considered. Studies were included if they focused on clinical samples from companion animals and provided detailed information on sampling techniques relevant to bacterial identification in dogs or cats. To complement the database search, additional studies were identified through citation searching and reference lists of relevant review articles.

The exclusion criteria were: articles not written in English; studies involving species other than dogs and cats; studies that did not describe sampling methods or that investigated non-clinical samples, such as environmental or food sources; studies focused on non-bacterial organisms (such as fungi or parasites); publications for which full-text access was not available through the university’s institutional subscriptions; and publications comprising scientific meeting abstracts, the literature reviews, short communications or case reports.

### 2.2. Selection Process and Data Extraction

All search results were exported to Microsoft Office™ Excel. Results from the initial search were evaluated according to the inclusion criteria. First, the results were screened by reading the article titles and excluding articles that were not relevant according to the inclusion criteria. Afterwards, the study abstracts were evaluated, and non-relevant articles were excluded. Subsequently, the selected full-text articles were retrieved and assessed according to the inclusion criteria. Any disagreements regarding study eligibility were resolved through discussion and consensus among the authors. After final selection, the following data were extracted from each selected study to elaborate a systematic database: article title, authors, article type, target animal species, origin and type of samples, method of sample collection, sample conservation method and time elapsed between collection and processing, study objectives, and information on antibiotic use prior to sample collection.

When a study described more than one sample type, collection method, or storage condition, each instance was recorded as an independent data point. This approach ensured that all sample-specific recommendations were captured, even when derived from a single publication. Consequently, the total number of data entries exceeded the total number of studies included in the review. For instance, storage and transport condition records amounted to 54 for aerobic cultures, 24 for anaerobic cultures, and 4 for *Campylobacter* cultures. Relative frequencies were calculated to describe how often a given parameter or recommendation appeared across the included studies. Therefore, in Section 3.4, Section 3.5 and Section 3.6, results are presented as proportions of the total number of records.

### 2.3. Risk of Bias Assessment

Risk of bias (RoB) was assessed using different critical appraisal tools to ensure a comprehensive and study type-specific evaluation of the methodological quality of the included studies, considering the specific characteristics of each study type [13]. Accordingly, the Joanna Briggs Institute (JBI) Critical Appraisal Tool was used for cross-sectional, prevalence, and expert opinion studies; the QUADAS-2 tool was applied for diagnostic accuracy studies; and the AGREE II tool was employed for clinical practice guidelines. The RoB of each study was independently analysed. No software was used; the evaluation was conducted manually based on the criteria suggested by each critical appraisal tool.

For the JBI tool, the evaluation focused on the clarity of inclusion criteria, detailed descriptions of the setting and participants, validity and reliability of sampling methods, appropriateness and justification of sample size, standardisation and reliability of measurements, management of confounding factors, clear reporting of sample handling, consistency and objectivity of outcome measurement and appropriateness of statistical analysis (Table A1) [14]. The QUADAS-2 tool examined potential biases related to patient selection, index test, reference standard, and flow and timing, as well as overall applicability concerns (Table A2) [15]. The AGREE II instrument assessed guideline quality across six domains: scope and purpose, stakeholder involvement, methodological rigour, clarity of presentation, applicability, and editorial independence (Table A3) [16].

For all tools, the following scoring criteria were applied: Y = Yes, N = No, U = Uncertain and N/A = Not applicable. An overall RoB score was calculated by assigning one point to each “Yes” response and zero points to each “No” or “Uncertain” or “Not applicable”. The total score was converted to a percentage of criteria met, allowing studies to be classified as low risk (≥80% of items met), moderate risk (50–79%), or high risk (<50%) [17].

## 3. Results

### 3.1. Description of Studies

**Figure 1:**
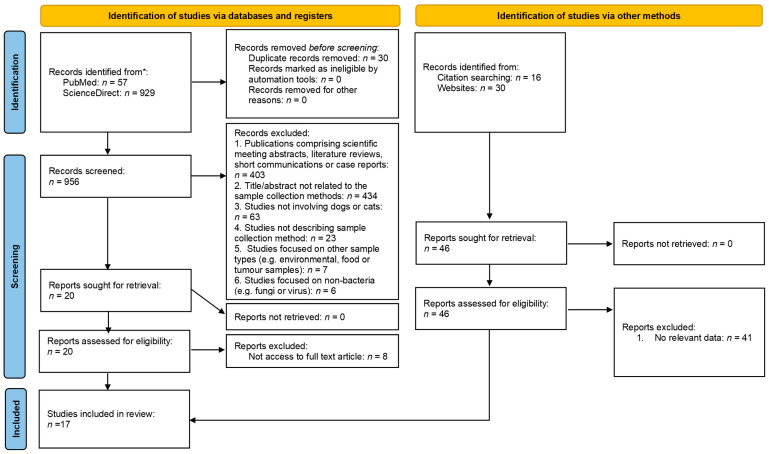
PRISMA 2020 flow diagram. * Consider, if feasible to do so, reporting the number of records identified from each database or register searched (rather than the total number across all databases/registers). Adapted from [11].

The PRISMA flow diagram provides an overview of the screening and selection process (Figure 1), while the characteristics and methodological quality of the included studies are detailed in Table 1. The initial search retrieved 986 records, of which 30 duplicates were removed, leaving 956 records for screening. Following title and abstract screening, 936 records were excluded. The full texts of the remaining 20 studies were then assessed for eligibility, resulting in the exclusion of 8 studies. In addition, five studies were identified through other methods and assessed for eligibility. In total, 17 studies met the inclusion criteria and were incorporated into this systematic review.

### 3.2. Quality Assessment

For cross-sectional, prevalence, and expert opinion studies (*n* = 10), most studies showed clear inclusion criteria, valid sampling methods, and consistent outcome measurement, while some uncertainty was noted regarding confounding factors and sample handling (Table A1). Overall, the risk of bias was rated low in four studies and moderate in six.

All diagnostic accuracy studies (*n* = 4) adequately addressed potential biases related to patient selection, index test, reference standard, flow and timing, and overall applicability concerns (Table A2). Consequently, all studies were rated as low risk of bias.

Among clinical practice guidelines (*n* = 3), scope, purpose, clarity of presentation, and applicability were generally well reported (Table A3). In contrast, stakeholder involvement, methodological rigour, and editorial independence were inconsistently described. Overall, one guideline was rated as low risk of bias, while two were rated as moderate.

Considering this overall assessment, the included studies were classified as presenting a low [20,21,24,25,26,28,29,30,32] to moderate [10,18,19,22,23,27,31,33] risk of bias and can therefore be regarded as methodologically acceptable for inclusion.

### 3.3. Characteristics of Studies and Outcome Measures

The characteristics of the included studies are summarised in Table 1. Of the 17 studies reviewed, 13 studies focused on sample collection in dogs and cats [18,20,21,22,24,25,26,27,28,29,31,32,33], two were limited to cats [19,30], one included dogs, cats, and exotic species [23], and one encompassed both companion and livestock animals [10].

With respect to sample types, eight studies investigated urinary tract samples [10,20,22,23,24,26,32,33], five skin samples [10,19,23,24,30], five respiratory tract samples [10,21,23,24,28], five faecal samples [10,23,24,30,31], four blood samples [10,23,24,33], four body cavity effusions [10,22,24,25], three synovial fluid samples [10,23,24], three tissue samples [10,22,23], two cerebrospinal fluids (CSF) [23,24], two ocular samples [18,23] and two reproductive tract samples [10,24]. In addition, single studies reported on other sample types, including bile [29], oral mucosa [30], bone marrow [23] and nail [23].

Regarding sampling methods for subsequent microbiological analysis, swabs were reported in nine studies [10,18,19,21,23,24,27,28,30], cystocentesis in seven [20,22,23,24,26,32,33], biopsy in five [10,22,23,24,28], aspiration in five [10,18,22,23,24], midstream urine collection in four [20,22,23,32], venipuncture in four [10,23,24,33], manual faecal collection in three [10,23,31], urinary catheterisation in three [22,23,32] and bronchoalveolar lavage (BAL) in three [10,23,24]. Less frequently reported techniques, each mentioned in a single study, included thoracocentesis [25], indwelling thoracic catheter [25], cholecystocentesis [29], spatula sampling [18], uterine lavage [24], manual milk extraction [24], semen collection [24], lumbar puncture [23] and ventricular aspiration [23], as well as tracheal washes [10] and blade collection [23].

### 3.4. Collection Methods and Specifications

The collection methods and specifications are summarised in Table 2.

#### 3.4.1. Body Cavities: Reported Data

Effusion samples (*n* = 3) were mainly collected by aspiration, with one study reporting collection either by aspiration or swab. For aspirated samples, two studies reported placing the material in plain sterile plastic containers, while one recommended transferring the aspirated content onto a swab placed in transport medium.

#### 3.4.2. Central Nervous System: Reported Data

Sampling was reported in two studies, both describing cerebrospinal fluid (CSF) collection. One study reported CSF collection by aspiration, while the other described either ventricular aspiration or lumbar puncture. Sterile tubes were recommended in both studies, with one study specifying the use of an aseptic subdural tap.

#### 3.4.3. Circulatory System: Reported Data

Five studies reported sampling from blood (*n* = 4) and bone marrow (*n* = 1). For blood collection, three studies sampled from the jugular vein, and three studies used specific blood culture bottles. Volumes of blood collected varied with animal weight, ranging from 1.5 to 10 mL for microbiological analysis. Bone marrow collection was recommended by aspiration into a sterile plain container.

#### 3.4.4. Gastrointestinal (GI) System: Reported Data

Eight studies reported sampling from the oral mucosa (*n* = 1), bile (*n* = 1), faeces (*n* = 5), and perianal area (*n* = 1). Oral mucosa sampling was performed using cotton swabs placed in transport media. Bile samples were collected through ultrasound-guided percutaneous cholecystocentesis. For faeces, four studies employed manual collection and two used swabs (with one study reporting both). Manually collected samples were placed in faeces-specific transport media (*n* = 2) or sterile containers (*n* = 2), with an approximate amount of 5 g per sample. Swabs were typically placed in Amies or Stuart transport medium. Perianal sampling was reported in a single study using a swab.

#### 3.4.5. Integumentary System: Reported Data

Abscesses or wounds (*n* = 3), crusts (*n* = 1), epidermal collarettes (*n* = 1), external ear canals (*n* = 3), middle ear (*n* = 1), nails (*n* = 1), pustules (*n* = 1), pyoderma (*n* = 1), skin (*n* = 4) and surgical sites (*n* = 1) were described in the selected studies. Swab collection was the most common method for abscesses and wounds, with aspiration also reported in two studies. Crusts, epidermal collarettes, and surgical site samples were each collected by swab in a single study. External ear canals were sampled by swab in all three studies, while middle ear collection involved myringotomy using a stiff cat urinary catheter with a syringe. Nail samples were collected using a blade or swab; pustules by aspiration; and pyoderma by biopsy. Skin samples were consistently collected by swab (*n* = 4). Among swab-collected samples (*n* = 14), ten studies recommended placement in transport medium. Aspirated material (*n* = 4) was generally transferred to a swab in transport medium.

#### 3.4.6. Musculoskeletal System: Reported Data

Sampling was reported in seven studies, including tissue (*n* = 3), post-mortem tissue (*n* = 1), and synovial fluid (*n* = 3). A biopsy was recommended for tissue and post-mortem samples. The samples were placed in plain sterile containers (*n* = 3), tubes containing transport medium (*n* = 1), or tubes with a clot activator (*n* = 1). One study suggested placing tissue in sterile tubes with a small amount of buffered solution, while another advised gently pressing the sample just below the surface of the medium, discarding the swab, and avoiding full submersion. Synovial fluid was collected by aspiration in all studies, with one also reporting swab collection. Equipment varied across studies, including sterile tubes, blood culture bottles, sterile containers, syringes, or swabs with transport medium.

#### 3.4.7. Ocular System: Reported Data

Four studies reported ocular sampling, using swabs (*n* = 2), a spatula (*n* = 1), or aspiration (*n* = 1).

#### 3.4.8. Reproductive System: Reported Data

Only one study described sampling from the vagina, uterus, mammary gland, testicles, prostate, and semen. Methods included biopsy or uterine washes, manual milk extraction, collection of the second or mid-third fraction of the ejaculate, rectal prostatic massage, cystocentesis, and swab sampling, respectively. Most samples were collected using sterile containers, except for the swab samples, which required a transport medium.

#### 3.4.9. Respiratory System: Reported Data

Five studies described upper airway sampling and three lower airway sampling, with one study addressing pleural fluid. Upper airway samples included the nostrils, the nasal cavity, the mucosa, and the sinuses. Swabs were used in five studies and biopsies in three. Swabs were placed in Amies transport medium in four studies and moistened prior to sampling in one. Biopsies were performed blindly (*n* = 1) or under endoscopic guidance (*n* = 2). Lower airway samples were obtained exclusively by bronchoalveolar lavage (*n* = 3), with tracheal wash and bronchial brush reported in one study each. Pleural fluid was collected by thoracocentesis or an indwelling thoracic catheter into non-anticoagulant tubes using aseptic technique.

#### 3.4.10. Urinary System: Reported Data

Thirteen studies reported urine sampling, most frequently by cystocentesis (*n* = 7), followed by catheterisation (*n* = 3) and midstream catch (*n* = 3). Of the studies using cystocentesis, two specified ultrasound-guided collection. Urine obtained by cystocentesis was collected in plain sterile containers (*n* = 4) or boric acid tubes (*n* = 2). Catheterised and midstream samples were primarily collected in plain sterile containers. The volume of urine collected varied across methods, ranging from 3 to 6 mL.

### 3.5. Consideration of Antibiotic Therapy Before Sampling

Among the 17 selected studies, three merely noted whether the animals had received antibiotics at the time of sampling. Two studies required a four-week antibiotic withdrawal period, and two specified a three-day withdrawal period. One study explicitly stated that ongoing antibiotic treatment should be reported on the sample submission form, and one indicated that sampling should be performed before the initiation of antibiotic therapy. The remaining eight studies provided no information regarding antibiotic administration (Table 3).

### 3.6. Target Bacterial Groups and Sample Storage Conditions Across Biological Systems and Sample Types

Across biological systems, most studies recommended testing for both aerobic and anaerobic bacteria, with a few notable exceptions driven by anatomical site or expected pathogens (Table 4). Body cavities effusions and CSF consistently required investigation of both groups. Similarly, blood, bone marrow and the musculoskeletal system generally followed this pattern. Notably, for post-mortem tissue, one study suggested testing for aerobes and/or anaerobes, as well as for *Campylobacter jejuni*, *Yersinia*, and *Salmonella.*

In contrast, skin samples frequently targeted only aerobes. This was evident in studies involving oral mucosa, external ear samples, nails, surgical sites, and several integumentary samples. Conversely, abscesses, wounds, and deeper integumentary infections typically included both aerobes and anaerobes.

Within the musculoskeletal, reproductive, and respiratory systems, recommendations varied, reflecting differences in anatomical depth and likelihood of anaerobic involvement. Upper airway samples, ocular and urine samples largely focused on aerobes, whereas lower airway and pleural samples included anaerobes more often. Semen was one of the few reproductive samples where anaerobic investigation was also suggested.

Storage conditions also varied by the bacterial group targeted. For aerobic bacteria, refrigeration was the most common recommendation, typically for up to 24–48 h, though some studies permitted longer or unspecified durations (Table A4). A minority emphasised rapid processing within 8 h. For anaerobic bacteria, recommendations tended to favour room-temperature storage, often up to 24–72 h. Reports targeting *Campylobacter* spp. showed no clear consensus, with both refrigeration and room temperature storage being described.

## 4. Discussion

The accuracy of microbiological diagnostics in veterinary practice depends primarily on the quality of the sample collected. Even when advanced laboratory techniques are available, inappropriate sampling tools, suboptimal transport conditions, or poor handling can compromise specimen integrity and reduce diagnostic accuracy [10,34]. In companion animal practice, where infections often require rapid and targeted antimicrobial therapy, the consequences of inadequate sampling include misleading culture results, delayed treatment, and a potential contribution to antimicrobial resistance [35,36]. Despite this critical role, veterinary-specific guidance on collection and storage remains limited and heterogeneous, highlighting the need for standardised, evidence-based practices.

In this systematic review, 17 articles were included to assess how sample collection methods, handling procedures, and transport conditions influence the reliability of microbiological results in dogs and cats. The findings revealed considerable variability, with potential implications for diagnostic accuracy and reproducibility.

### 4.1. General Patterns and Implications for Diagnostic Reliability

Across all biological systems, swab collection emerged as the most frequently reported method [37,38]. While swabs are convenient and minimally invasive, they are also more prone to contamination, yield limited sample volumes, and are less likely to capture deep or truly representative material from infected tissues [38,39].

Tissue biopsies demonstrate the highest diagnostic yield, with superior sensitivity, specificity, and predictive values, particularly for periprosthetic joint infections and infected diabetic foot ulcers [40,41]. In situations where biopsy is not feasible, pus aspirates provide a less invasive alternative, enabling the collection of diagnostically relevant material while acknowledging their lower sensitivity compared with tissue biopsy [38]. Nevertheless, the predominance of swab use, even in body sites where aspiration or biopsy would be more appropriate, may be driven more by logistical considerations than by evidence-based criteria. A similar pattern has been reported in human medicine, where pre-analytical errors remain a major contributor to diagnostic inaccuracy, often exceeding those arising from analytical or post-analytical phases [42].

### 4.2. Sampling Methods by Organ System

#### 4.2.1. Body Cavities, the Central Nervous and Circulatory Systems

**Figure 2:**
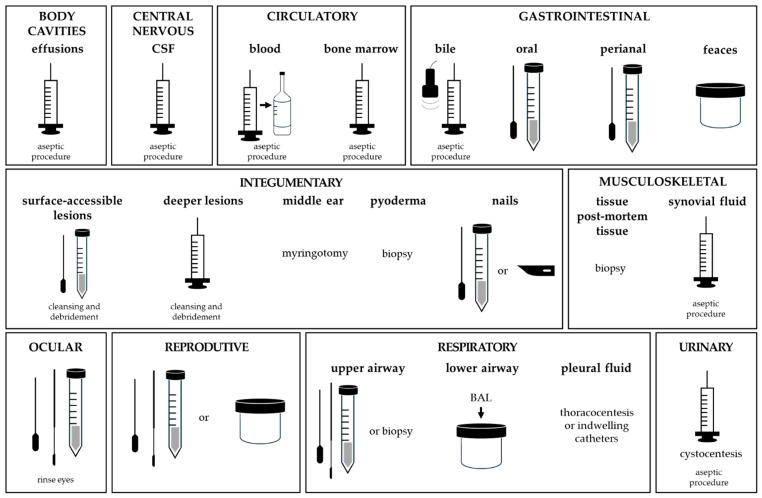
Overview of microbiological sample collection methods.

For cerebrospinal fluid (CSF) and body cavity effusions, aspiration was consistently used, reflecting good procedural standardisation. CSF samples were collected using aseptic subdural tap and sterile tubes, following recommendations similar to those in human medicine [43]. When immediate culture is not possible, CSF may alternatively be submitted in blood culture vials to improve microorganism recovery during delayed processing (Figure 2) [44].

Blood sampling appeared relatively uniform, with jugular venipuncture and blood culture bottles reported as standard. Sample volumes were generally modest, typically around 2.5 mL per bottle, or adjusted according to the animal’s weight, particularly in dogs. Although these volumes are lower than those recommended in human protocols, diagnostic sensitivity may still be compromised in cases of very low bacteraemia. Similar observations have been reported in human medicine, where insufficient blood volumes can lead to false-negative results, delayed therapy, and increased morbidity [45,46,47]. The sensitivity of blood cultures is directly correlated with the volume collected, as microbial loads in bloodstream infections may be as low as 1–10 CFU/mL [46,48]. Adult human protocols recommend 40–60 mL distributed across multiple bottles to optimise pathogen recovery, but such volumes are impractical in veterinary settings due to the wide variability in body sizes and circulating blood volumes [45]. Furthermore, contamination with commensal skin microorganisms during sample collection remains a significant cause of false-positive results, leading to unnecessary antibiotic treatment and extended hospitalisation [47]. As bloodstream infection–associated pathogens may originate from the skin and bacterial loads in blood can be as low as 0.25 CFU/mL, minimising contamination collection and preserving pathogen viability during transport to the laboratory are critical for accurate diagnosis [44].

Bone marrow sampling (BMS), reported in only one study, was performed by aspiration under aseptic conditions, in accordance with currently accepted best practices [49]. BMS provides valuable diagnostic informations for haematological and infectious diseases, such as *Leishmania*, feline leukaemia virus (FeLV), and Histoplasmosis [50]. Despite being minimally invasive, it remains underutilised in clinical practice, often due to misconceptions regarding technical difficulty and the perceived need for specialised equipment [49].

#### 4.2.2. Gastrointestinal System

Sampling within the gastrointestinal system ranged from the oral mucosa and bile to faeces and perianal sites. Ultrasound-guided cholecystocentesis was used for bile, reflecting high technical standard [51]. Faecal samples were typically collected manually, whereas swabs were commonly used for oral mucosa and perianal sites. Swab-based collection may limit bacterial representation due to smaller sample volumes. However, modern swab, such as flocked swabs with dedicated transport media, have substantially improved culture recovery and overall specimen quality [52].

Rectal swabs, in particular, have been highlighted as a practical alternative to bulk faecal samples. Their ease of collection and transport supports surveillance of multidrug-resistant organisms (VRE, CRE) [53]. In addition, swabs have demonstrated comparable sensitivity to faecal specimens for enteric bacterial culture, and recent PCR-based studies indicate equivalent diagnostic performance for *Clostridium difficile* infection, enabling faster turnaround times and improved infection control [54]. Despite their practicality, swabs still pose several limitations. In humans, inadequate contact with mucosal surfaces can result in false-negative results, particularly in self-sampling contexts. Furthermore, rectal swabs have shown lower sensitivity than stool for real-time PCR detection of *Giardia duodenalis*, performing optimally only at higher parasite loads [55,56]. Nevertheless, multiple studies comparing rectal swabs and faecal samples in microbiota research have demonstrated strong correlations in bacterial community composition, diversity, and inferred functional pathways, with additional metabolomic analyses confirming high concordance across most metabolites [57,58].

Overall, rectal swabs, especially when collected using flocked designs and appropriate transport systems, represent a valid and efficient alternative to faecal samples for bacterial culture, molecular diagnostics, and microbiota profiling. Their use should, however, take into account sampling adequacy, reduced volume of collected material, and potential variation in sensitivity across different target organisms.

#### 4.2.3. Integumentary System

Samples from the integumentary system encompassed the widest diversity of specimen types in this review, including abscesses or wounds, crusts, epidermal collarettes, external and middle ear canals, nails, pustules, pyoderma, general skin lesions and surgical sites. Prior cleansing and debridement is essential to minimise contamination and recover true pathogens rather than surface colonisers [59].

For surface-accessible lesions, such as crusts, epidermal collarettes, the external ear canal, skin in general and surgical sites, swab collection remains the most recommended and practical because of its ease of use and minimal invasiveness [60]. Moreover, comparative studies in human skin-microbiome research indicate that swabs effectively capture superficial microbiota, although the yield of viable bacteria may be lower than that obtained with deeper sampling methods [61].

In contrast, for lesions extending into deeper tissue (such as abscesses, wounds with pus, or pustules), fine-needle aspiration is preferred, as it allows direct access to the focus of infection while minimising surface contamination [59]. Interestingly, our review identified several studies that recommended transferring aspirated material onto a swab before submission. However, this practice may inadvertently reintroduce surface contamination and undermine one of the principal advantages of aspiration, particularly direct sampling of the lesion core. Therefore, this approach warrants caution in the context of veterinary dermatologic microbiology protocols.

For middle ear sampling, myringotomy is the recommended procedure, as it provides access to the middle ear exudate while minimising contamination from the external ear canal. This procedure is particularly relevant in chronic or refractory cases of otitis media, in which cultures obtained directly from the middle ear cavity yield more representative microbiological data [62].

In cases of pyoderma, biopsy sampling is advised. In human medicine, studies comparing superficial swabs and deep-tissue biopsies in skin and soft tissue infections have shown that biopsies offer greater sensitivity and more accurate pathogen identification [63]. This finding supports similar recommendations for veterinary pyoderma, particularly when empirical therapy fails or when a deep-seated infection is suspected.

Finally, for nail samples, the choice between blade scraping or swabbing depends on the lesion type and the suspected pathogen. For superficial nail plate colonisation, a moistened swab may be sufficient, whereas for subungual or peri-ungual involvement, scraping with a sterile blade yields better diagnostic material [64].

#### 4.2.4. Musculoskeletal System

Tissue and post-mortem tissue, as well as synovial fluid were included. Biopsy collection was the preferred method for tissue, with specimens commonly placed in plain tubes or tubes containing a clot activator [65]. In addition, partially immersion of tissue specimens in transport medium represents good practice, as it helps to prevent desiccation and preserves the viability of infectious agents during transport and storage [66].

Synovial fluid should be aspirated aseptically [67], handled under sterile conditions to minimise contamination [67]. For microbiological analysis, transfer of the sample into plain containers is recommended, as EDTA interferes with bacterial growth and may compromise culture results [68]. Alternatively, direct inoculating into blood culture bottles has been shown to enhance microbial detection and overall diagnostic yield [69]. Nevertheless, transferring aspirates onto swabs is discouraged due to contamination risks, as discussed previously.

#### 4.2.5. Ocular System

For conjunctival sampling, several techniques have been described in the selected studies. Before sample collection, when dyes or topical anaesthetics have been applied, the ocular surface should be thoroughly rinsed with sterile saline or sterile water to ensure optimal detection of ocular pathogens [70]. In human medicine, conjunctival samples are typically obtained using a sterile ocular swab, which is finer, softer and less traumatic, particularly suitable for small animals [71,72]. These swabs are commonly pre-moistened with sterile saline and gently rolled over the lower bulbar conjunctiva or fornix and, when indicated, along the lid margin, while carefully avoiding contact with the eyelashes and cornea [70]. Notably, flocked swabs have been shown to enhance cellular and microbial recovery when compared with conventional cotton swabs [73].

Consistent with these findings, in a study investigating the ocular surface microbiome, the lower conjunctiva was swabbed three times using either a flocked or a cotton swab, with flocked swabs yielding a higher microbial recovery, supporting their preferential use for ocular surface sampling [74]. Following collection, the swab is either placed directly into an appropriate transport medium or into a sterile dry tube, showing no significant differences if processed promptly [70,75]. It has also been recommended that, even in cases of unilateral disease, samples should be collected from both eyes (infected and non-infected) to help differentiate true pathogens from commensal flora [70].

#### 4.2.6. Reproductive System

Reproductive sampling was sparsely reported, despite its clinical relevance in breeding animals. The two studies addressing this system described a wide range of sample types, including vaginal, uterine, and mammary gland samples, as well as prostate and semen collections. Most specimens were placed in sterile containers, whereas the use of swabs was recommended only for semen sampling.

Evidence from human medicine underscores that, when tissue or fluid is collected from deeper reproductive organs, such as the uterus or endometrium, strict aseptic conditions and careful handling are essential to minimise contamination, which could otherwise compromise data interpretation [76]. Similarly to ocular sampling, flocked swabs may also be employed for reproductive tract specimens, as their design allows enhanced recovery of epithelial cells and microorganisms while minimising trauma, and their use has already been reported in studies of the female reproductive tract [77]. Semen sampling for microbiome or fertility assessment in human medicine typically involves collecting the entire ejaculate into a sterile container following a period of abstinence, ensuring appropriate genital hygiene, and maintaining controlled time and temperature conditions before processing [78].

#### 4.2.7. Respiratory System

Upper airway samples (ranging from the nostrils to the sinuses) were most frequently obtained using swabs or biopsies performed under rhinoscopic guidance. Flocked swabs may also be suitable for sample collection, as previously described for ocular and reproductive sampling [79]. This approach is considered practical because it is minimally invasive, allows targeted sampling of relevant mucosal sites, and has demonstrated good diagnostic performance in human medicine, where upper respiratory swabs show pooled sensitivity and specificity values of approximately 91% and 98%, respectively, for pathogen detection [80].

In contrast, sampling of the lower airways typically relies on bronchoalveolar lavage (BAL), tracheal wash, or bronchial brush techniques. BAL is widely regarded as the gold standard for lower respiratory tract sampling because it minimises contamination from upper airway flora and provides a high diagnostic yield [81]. A recent review in paediatric human patients further supports the diagnostic yield and safety of BAL, highlighting potential parallels with companion animal practice [82]. The procedure involves advancing the bronchoscope into the bronchopulmonary segment of interest until wedged, followed by instillation of 100–300 mL of sterile saline, with at least 30% of the instilled volume retrieved for optimal analysis [81]. The sample should be collected in a labelled sterile container and transported promptly to the laboratory [81]. However, some comparative studies have reported that tracheal aspirates and BAL fluid may exhibit similar microbial diversity, suggesting that less invasive approaches can still yield representative results in certain clinical contexts [83].

Notably, two of the three reviewed studies recommended transferring the aspirate onto a swab after collection. However, this practice may increase the risk of contamination and compromise downstream microbiological analyses. In line with human medical protocols, lower airway aspirates should instead be placed directly into a sterile container to preserve sample integrity [84].

Additionally, pleural fluid samples were collected via thoracocentesis or through indwelling catheters under aseptic conditions, using non-anticoagulant tubes. Although these recommendations were reported in only one of the reviewed studies, this approach represents best practice [85].

#### 4.2.8. Urinary System

The urinary system was the most frequently investigated, showing relatively good alignment with established best practices. Cystocentesis was the preferred collection method, reflecting awareness of its greater reliability in reducing contamination when compared with midstream voided samples or catheterisation [86,87]. Although two studies reported the use of preservatives such as boric acid and four referred to the use of sterile containers, plain sterile containers remain appropriate for urine collection when samples can be promptly refrigerated and analysed within the recommended time frame (Section 4.4) [88,89]. Consequently, the choice between a plain sterile container and a tube containing a preservative is primarily determined by the expected storage and transport conditions (Section 4.5).

### 4.3. Impact of Antimicrobial Therapy Before Sampling

Consideration of antibiotic exposure prior to sample collection was notably inconsistent across the seventeen included studies. While a few studies defined withdrawal periods (either 4 weeks or 72 h) or recorded whether animals had received antibiotics, others required formal reporting of ongoing therapy or recommended sampling before antibiotic initiation. However, nearly half of the studies did not report any information regarding prior or ongoing antibiotic use; four were cross-sectional studies, and one was a case report, in which antibiotic exposure would not have been relevant given the study design and scope. This lack of standardisation represents a significant methodological limitation, particularly considering the well-documented suppressive effect of antibiotics on bacterial recovery [90]. Even short-term systemic antimicrobial exposure can exert a post-antibiotic effect, transiently inhibiting bacterial regrowth and thereby reducing culture sensitivity [91,92]. For example, in respiratory sampling, BAL, despite being considered the gold standard, has been shown to exhibit markedly reduced sensitivity following antimicrobial treatment, especially for fastidious microorganisms. In such cases, molecular assays such as PCR have proven to be valuable complementary diagnostic tools [81]. In both clinical and research contexts, failure to account for prior antibiotic exposure may result in false-negative culture results or underestimation of antimicrobial resistance prevalence [93,94]. Beyond their immediate microbiological impact, antibiotics can induce profound and long-lasting alterations in host-associated microbiota, reducing microbial diversity and disturbing ecological balance [95]. These dysbiotic shifts can persist for months or even years, depending on the drug spectrum and duration of treatment, thereby weakening colonisation resistance and facilitating opportunistic infections, such as Clostridium difficile and *Salmonella* Typhimurium [96]. Collectively, these effects highlight the broader consequences of antibiotic exposure and underscore the importance of accurate antibiotic use, as well as the adoption of precision veterinary medicine approaches in which therapeutic decisions are guided by culture and susceptibility testing, thereby reinforcing antibiotic stewardship in veterinary practice [96]. Therefore, whenever clinically feasible, samples should ideally be collected before starting antibiotic therapy. When this is not possible, sampling should be delayed until at least seven days after the last antibiotic dose, particularly in cases involving chronic or deep-seated infections or when long-acting antibiotics have been administered [23,97,98]. In such situations, complementing culture with molecular methods (e.g., PCR or metagenomic sequencing), together with careful documentation of prior antibiotic exposure, becomes essential to ensure accurate interpretation of results [97].

### 4.4. Bacterial Target

**Figure 3:**
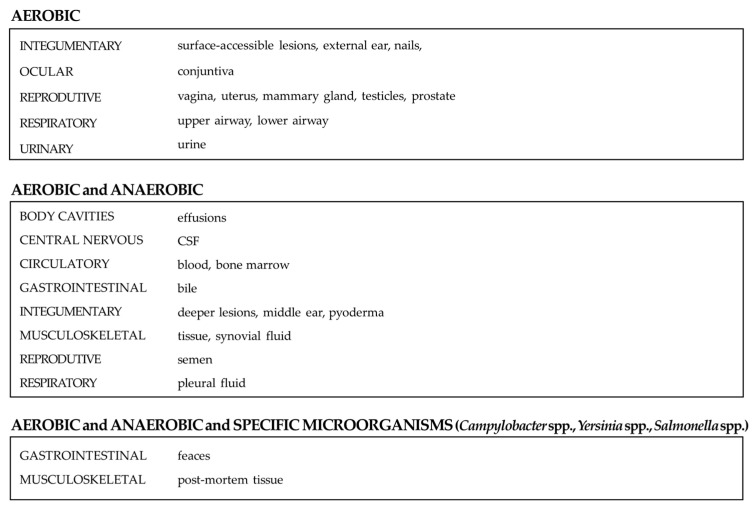
Overview of bacterial targets by sample type and anatomical origin.

The bacterial groups investigated varied according to the sample type and its anatomical origin (Figure 3). In general, cultures targeting aerobic bacteria were recommended for specimens obtained from sites naturally exposed to atmospheric oxygen, such as oral mucosa, external and middle ear, nails, surgical sites, conjunctiva, vagina, uterus, mammary gland, testicles, prostate, upper and lower airways, and urine. This approach is consistent with standard practices in human clinical microbiology [99,100].

With regard to urine sampling, although some authors advocate investigation of anaerobic bacteria in suspected cases of emphysematous cystitis, this condition is uncommon and is usually caused by aerobic, gas-producing bacteria, including *Escherichia coli* (the predominant pathogen), *Klebsiella* spp. and *Proteus* spp. [101,102]. Anaerobes such as *Clostridium perfringens* are only rarely implicated in urinary tract infection, and it is only in these exceptional cases that routine aerobic culture may be insufficient [103].

In contrast, samples derived from body cavity effusions, bile, abscesses, wounds, crusts, epidermal collarettes, pustules, pyoderma and pleural fluid should include investigation for both aerobic and anaerobic organisms, as these sites are frequently infected by mixed bacterial populations [99,100,104]. Similarly, for cerebrospinal fluid, blood, faeces, tissue, synovial fluid, and semen, combined aerobic and anaerobic testing is advisable, given that anaerobic pathogens can occasionally cause infections at these sites [99,105,106,107]. Post-mortem tissue samples additionally require targeted investigation for *Campylobacter jejuni*, *Yersinia* spp., and *Salmonella* spp., as well as faecal sampling, mirroring established protocols in human medicine [108].

In summary, superficial or air-exposed samples typically warrant aerobic cultures alone, whereas samples obtained from deeper or normally sterile anatomical sites (e.g., inner ear, blood, body cavity effusions, and deep abscesses) should prompt consideration of both aerobic and anaerobic culture strategies.

### 4.5. Storage and Transport Conditions

**Figure 4:**
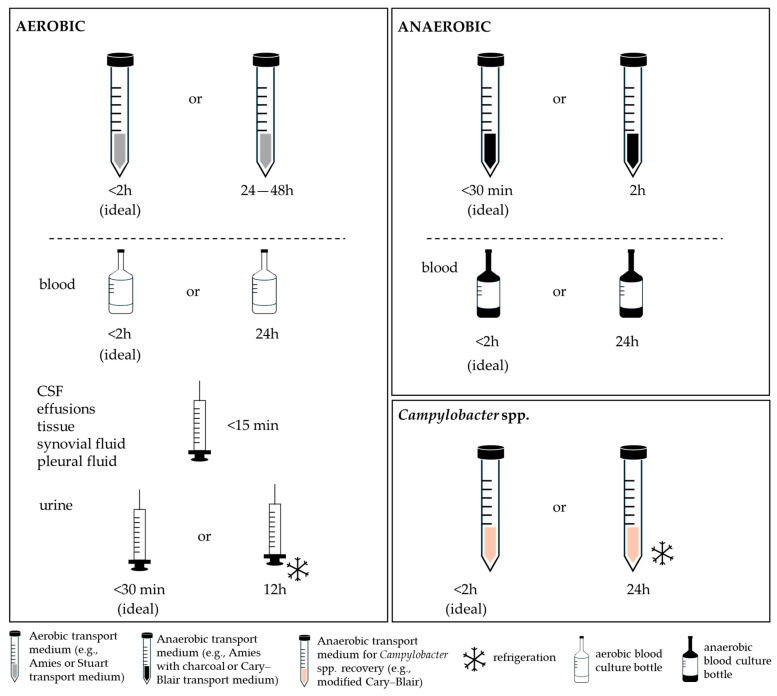
Overview of storage and transport conditions according to the bacterial target.

Storage and transport conditions were inconsistently described across the included studies, with critical details such as temperature and duration often omitted (Figure 4). For aerobic bacteria, refrigeration at approximately 4 °C for up to 24–48 h was the most commonly recommended condition and is consistent with general microbiological standards [109]. Ideally, samples should be transported to the laboratory within two hours of collection [99]. Bayley and colleagues further emphasise that for certain samples, such as body cavity effusions, CSF, and biopsy tissues, the interval between collection and processing should not exceed 15 min if refrigeration or appropriate transport media are not available [99]. These authors also note that when immediate processing upon arrival is not possible, specimens must be stored under suitable conditions; for example, urine, faeces, sputum, swabs, and indwelling devices (e.g., catheters) should be kept at 4 °C, whereas tissues requiring long-term storage should be frozen at −70 °C [99].

Beyond these general principles, several specimen-specific considerations emerged. Human urinalysis guidelines indicate that when immediate examination (within 30 min) is not feasible, urine should be refrigerated at 4 °C and analysed within 12 h to limit bacterial proliferation [88]. If refrigeration is not feasible, containers pre-filled with preservatives such as boric acid may be used [110]. Boric acid can maintain leukocyte integrity and stabilise bacterial counts for up to 24 h at approximately 20 °C; however, its use may impair the recovery of certain bacteria, notably *Pseudomonas* spp. [110]. For ocular samples, particularly conjunctival swabs, standard ophthalmic microbiology protocols recommend immediate transport at room temperature. When delays are unavoidable, samples should be placed in an appropriate transport medium and/or refrigerated to prevent desiccation and preserve bacterial viability [70].

For blood samples, some studies indicate that no significant loss in yield occurs when blood cultures are stored for less than 24 h at 25 °C [111,112]. However, guidelines recommend a maximum pre-analytical interval of 2 h from collection to incubation [99,113]. Notably, each hour elapsed from blood collection to incubation has been associated with a decrease of 0.3% in the probability of a positive result, highlighting the importance of rapid processing to maintain diagnostic accuracy [113].

Temperature control is a critical determinant of sample integrity, as it minimises enzymatic activity and uncontrolled bacterial growth. This is typically achieved through short-term refrigeration (2–8 °C) or deep freezing (−80 °C) for long-term storage [114,115]. Nevertheless, several reports described storage at room temperature for up to 72 h, a practice known to reduce bacterial viability and alter microbial community structure, particularly in microbiome-targeted analyses [116,117].

With respect to anaerobic bacteria, approximately one-third of studies recommended maintaining samples at ambient temperature for 24–72 h, while others suggested refrigeration without specifying a time limit. Current guidelines, however, recommend that anaerobic specimens be transported to the laboratory as rapidly as possible (ideally within 30 min) to minimise oxygen exposure and preserve microbial viability. Specimens collected in anaerobic transport media may be safely processed within up to two hours of collection [118,119].

*Campylobacter* spp. were rarely discussed, despite their clinical and epidemiological relevance [120]. Refrigeration for up to 48 h was the most common recommendation. However, *Campylobacter* species are highly sensitive to environmental conditions, including dehydration, atmospheric oxygen, sunlight, and temperature fluctuations [120,121]. Extreme temperatures (>20 °C or <0 °C) and fluctuations should therefore be avoided, with short-term storage at 4 °C (±2 °C) recommended when immediate processing is not possible. When collected using swabs, commercially available transport tubes containing protective media, such as Amies or charcoal-based, are advised to prevent desiccation and oxygen toxicity, while faecal material should be shipped in appropriate transport media, such as Cary-Blair or modified Cary-Blair [120,121].

While the above recommendations pertain primarily to culture-based analyses, molecular and nucleic acid–based techniques introduce additional pre-analytical considerations. Approaches such as immunohistochemistry, PCR, and Next-Generation Sequencing (NGS) require careful handling to preserve nucleic acids [122]. Samples often need rapid cooling, immediate processing, or stabilisers, and transport containers must not interfere with amplification or sequencing [123]. Non-invasive specimens may be suitable for molecular assays even when unsuitable for culture [124]. Failure to adhere to these pre-analytical precautions can reduce assay sensitivity, compromise nucleic acid integrity, and distort microbial composition, particularly in low-biomass or complex samples [125].

Overall, the substantial variability observed in storage conditions and handling procedures across studies exposes a major methodological gap between ideal laboratory standards and field implementation. The development and integration of validated, evidence-based veterinary guidelines, drawing from human diagnostic microbiology and supported by experimental validation, will be essential to improving culture accuracy, maintaining sample integrity and ensuring reproducible results across clinical and research settings.

### 4.6. Clinical and One Health Implications

The consequences of suboptimal or inconsistent sampling extend far beyond laboratory performance. Inaccurate or unreliable culture results undermine confidence in microbiological diagnostics, increasing the likelihood that clinicians resort to empirical or broad-spectrum antibiotic therapy [126]. This practice not only compromises individual patient care but also contributes to the emergence and dissemination of antimicrobial resistance (AMR) across animal and human populations [127].

Standardised sampling practices should therefore be regarded not merely as a technical refinement, but as a cornerstone of veterinary antibiotic stewardship and One Health-oriented AMR mitigation strategies. By improving the reliability of diagnostic results, standardisation enables clinicians to prescribe antibiotics less frequently and more judiciously, with greater confidence in therapeutic decision-making [128].

Notably, stressful handling during sample collection can trigger neuroendocrine responses that modulate host immunity and directly influence bacterial growth and microbiota composition [129,130]. Consequently, inadequate stress management may compromise the timing, precision, and quantity of the collected specimen, while operator skill directly affects its representativeness and the microbial species identified [131].

In addition, environmental and operator-induced contamination further threatens sample integrity, as clinical environments and surfaces can harbour diverse bacterial species that may confound microbiological results, and guidelines for sample collection explicitly emphasise avoiding cross-contamination between animals, instruments, and surroundings to preserve sample integrity [132,133]. In low-biomass samples, contaminants introduced during sampling workflows may even outweigh the true microbial signal, underscoring the necessity of stringent contamination control measures [134].

Importantly, bacterial identification in most studies is intrinsically linked to antibiotic susceptibility testing, which directly informs antibiotic choice. However, prior antimicrobial therapy can influence both the likelihood of pathogen recovery and the resulting susceptibility profile, potentially leading to false-negative cultures or biassed antibiograms [135,136]. Failure to account for such effects may therefore compromise both diagnostic accuracy and downstream clinical decisions.

**Figure 5:**
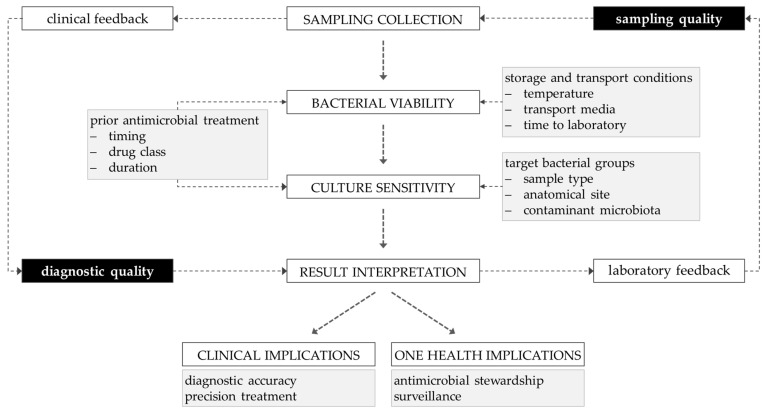
Post-collection workflow of microbiological samples in companion animals.

Beyond pre-analytical factors, rapid diagnostic tools can mitigate some limitations associated with suboptimal sampling [137]. Techniques such as MALDI-TOF and FTIR spectroscopy allow fast and accurate bacterial identification, often within hours, enabling clinicians to initiate targeted therapy quickly and reduce unnecessary antibiotic use, thereby limiting the development of multidrug-resistant organisms [138,139]. By complementing conventional culture and AST workflows, these technologies provide a bridge between sample quality and timely clinical decision-making, further supporting veterinary antibiotic stewardship and One Health objectives. Enhanced communication between clinicians and diagnostic laboratories is also essential. The adoption of standardised guidelines detailing appropriate sampling methods, storage, and transport conditions, together with explicit documentation of prior or ongoing antibiotic therapy, substantially enhances the interpretability and clinical relevance of culture and susceptibility results. Likewise, consistent laboratory feedback regarding specimen quality can foster continuous improvement in sampling practices and strengthen collaboration between clinical and laboratory teams (Figure 5).

## 5. Conclusions

This systematic review underscores the variability and existing gaps in microbiological sampling practices for companion animals. Inconsistent reporting of sample collection, handling, storage, and transport, along with limited documentation of critical factors such as prior antibiotic exposure, compromises the reliability of culture, diagnostic accuracy, and result interpretation. Although veterinary practice faces unique logistical and biological challenges, adopting and appropriately adapting evidence-based approaches from human diagnostic microbiology offers a promising path to improve outcomes. Overall, these findings highlight the urgent need for harmonised, species-specific, and evidence-based protocols that balance clinical feasibility with microbiological integrity. Strengthened collaboration between veterinary practitioners and diagnostic laboratories will be essential to translate these recommendations into routine practices, thereby supporting antibiotic stewardship and One Health objectives.

## Figures and Tables

**Table 1:** Characteristics of included studies after full assessment. Data from the final seventeen studies were extracted and systematically organised, including author, year of publication, article type, sample type, data collection method, and risk of bias (RoB) evaluation.

Author	Year	Species	Sample Type	Collection Method	RoB (%)	References
Athanasiou et al.	2018	dogs and cats	ocular	aspiration, spatulas, swabs	50	[18]
Cavana et al.	2023	cats	skin	swab	75	[19]
Coffey et al.	2020	dogs and cats	urine	cystocentesis and midstream catch	100	[20]
Elnageh et al.	2021	dogs and cats	nasal	swab	88	[21]
Franklin-Guil, et al.	2025	multiple animal species	blood, effusions, faeces, post-mortem tissues, skin, tissue, urine	aspiration, bronchoscopy-guided bronchoalveolar lavage (BAL), biopsy and hardware, manual collection of faeces, swab, tracheal wash, venipuncture	63	[10]
Gunn-Christie, et al.	2023	dogs and cats	effusions, tissue, urine	aspiration, biopsy, urinary catheterisation, cystocentesis, midstream catch	75	[22]
IDEXX Reference Laboratories	2025	dogs, cats and exotics	blood, bone marrow, cerebrospinal fluid (CSF), faeces, joint fluid, nail, nasal, ocular, skin, tissue and urine	aspiration, BAL, biopsy, blade, urinary catheterisation, cystocentesis, jugular vein puncture, lumbar puncture, manual faecal collection, midstream catch, swab, ventricular aspiration	67	[23]
Jessen, et al.	2018	dogs and cats	blood, effusions, faeces, reproductive tract, respiratory tract, skin and urine	aspiration, biopsy, BAL, cystocentesis, semen collection, jugular vein puncture, manual milk extraction, swab, uterine washes	100	[24]
Johnson et al.	2023	dogs and cats	pleural fluid	indwelling thoracic catheter, thoracocentesis	88	[25]
López-Córdova et al.	2025	dogs and cats	urine	cystocentesis	100	[26]
Menezes et al.	2022	dogs and cats	skin	swab	75	[27]
Niedenführ et al.	2024	dogs and cats	nasal	biopsy, swab	100	[28]
Pashmakova et al.	2017	dogs and cats	bile	cholecystocentesis	100	[29]
Rana et al.	2024	cats	oral, skin	swab	88	[30]
Torkan et al.	2018	dogs and cats	faeces	manual collection of faeces	75	[31]
Torre et al.	2022	dogs and cats	urine	urinary catheterisation, cystocentesis, midstream catch	100	[32]
Uva et al.	2024	dogs and cats	blood, urine	cystocentesis, jugular vein puncture	75	[33]

RoB: low risk (≥80%), moderate risk (50–79%), high risk (<50%).

**Table 2:** Sampling methods described in the selected studies, organised by organ system, sample type, collection technique, equipment, quantity and other relevant specifications.

Organ System	Sample Type		Collection Method	Equipment	Volume	Other Specifications	References
body cavities	effusions		swab or aspiration	swab with transport media or aspiration transferred to swab with transport media	-	swab saturated; add 2–3 mL of sample to gel medium; submit extra fluid in RTT	[10]
			aspiration	plain plastic container	-	-	[22]
			aspiration	sterile containers or syringes	-	-	[24]
central nervous	cerebrospinal fluid (CSF)		ventricular aspiration or lumbar puncture	WTT or RTT	-	aseptic subdural tap	[23]
			aspiration	sterile containers or syringes	-	-	[24]
circulatory	blood		jugular vein puncture	EDTA tubes	-	-	[24]
				BD Vacutainer^®^ Plus Citrate Plasma Tubes (Becton Dickinson, Franklin Lakes, USA)	2.5 mL	after aseptic skin preparation.	[33]
			venipuncture	blood culture bottle	-	-	[10]
				Oxoid Signal^®^ blood culture bottle (Oxoid Ltd, Basingstoke, UK; animals > 2 kg) or Wampole® Isolator™ blood culture tube (Oxoid; animals < 2 kg)	18 kg: 10 mL/84 mL bottle 9–18 kg: 7.5 mL/84 mL bottle 2–9 kg: 5 mL/84 mL bottle <2 kg: 1.5 mL/blood culture tube	aseptically prepare the venipuncture site; disinfect the top of the culture bottle with alcohol and let dry; one blood culture bottle per time point. Ideally, two samples drawn approximately one hour apart from different venous sites should be submitted.	[23]
	bone marrow		aspiration	WTT or RTT	-	aseptically prepare the collection site	[23]
gastrointestinal	oral mucosa		swab	cotton swab	-	-	[30]
	bile		cholecystocentesis	needle with 22-gauge, 1.5-inch needle with an attached 12 mL syringe	minimum volume of 1 to 3 mL	ultrasound-guided	[29]
	faeces		manual or swab	leak-proof faecal container or swab with transport medium	5 g	-	[10]
			manual	faecal culture transport medium (preferred) or sterile tube (WTT or RTT)	3–5 g	avoid samples that have had contact with soil for longer than a few minutes and avoid samples that have been sitting in cat litter.	[23]
			manual	sterile containers	-	-	[24]
			swab	transport medium *	-	-	
			manual	sterile plastic bags	5 g (minimum)	-	[31]
	perianal		swab	cotton swab	-	-	[30]
integumentary	abscess or wound		swab	swab with transport medium	-	saturate swab; add 2–3 mL fluid to gel medium; submit excess in RTT.	[10]
			swab or aspiration (pus)	culture swab in transport medium or in a sterile tube	-	aseptic preparation of the collection site	[23]
			swab or aspiration (pus)	swab in transport medium * or syringe and needle and transferred to a swab in transport medium *	-	no surface cleaning unless major contamination; drain tip with swab in the culture medium *	[24]
	crust		swab	swab in culture medium *	-	no surface disinfection; lift crust edge with sterile forceps and swab underlying skin	[24]
	epidermal collarette		swab	swab in culture medium *	-	no surface disinfection; trichotomy with sterile scissors, then swab the inner surface of the collarette.	[24]
	external ear canal		swab	swab in transport medium	-	topical treatments may inhibit bacterial growth.	[23]
			swab	swab in transport medium *	-	-	[24]
			swab	cotton swab	-	-	[30]
	middle ear		myringotomy	stiff cat urinary catheter coupled to a syringe; aspiration and transferred to sterile swabs in a transport medium *	-	-	[24]
	nail		blade or swab	sterile tube (WTT or RTT)	-	sterile blade or swab to collect material from the infected nail	[23]
	pustule		aspiration	sterile needle and transfer pus to swab in transport medium *	-	no surface disinfection; trichotomy with sterile scissors	[24]
	pyoderma		biopsy	punch with a diameter of 3–4 mm and place the biopsy in a sterile container moistened with one drop of sterile saline	-	anaesthesia or deep sedation; trichotomy with sterile scissors, disinfect skin with 70% ethanol; close biopsy site with staples or sutures.	[24]
	skin		swab	swab with transport medium	-	clean site with sterile saline and gauze; no antiseptics before sampling.	[10]
			swab	swabs with Amies transport medium	-	-	[19]
			swab	swab in a transport medium	-	scrape or swab the active border of skin lesions	[23]
			swab	cotton swab	-	-	[30]
	surgical site		swab	dry cotton-tipped swab	-	rubbed in the surgical site	[27]
musculoskeletal	tissue		biopsy or hardware	RTT or placed in a tube with transport medium *	-	press the sample just below the surface of the medium; discard the swab. Do not submerge. Add 0.5 mL sterile saline to RTT to keep tissue moist	[10]
			biopsy	sterile bags or tubes	-	-	[22]
			biopsy	WTT or RTT	-	place tissue in a sterile tube with a small amount of sterile buffered solution	[23]
	post-mortem tissue		biopsy	sealed, leak-proof container	>3 cm^3^	-	[10]
	synovial fluid		swab or aspiration	swab with transport medium or aspiration transferred to swab with transport medium	-	swab is saturated; add 2–3 mL of fluid to the gel medium; submit the extra fluid in a RTT.	[10]
			aspiration	sterile tube (WTT or RTT) or blood culture bottle.		inject fluid aseptically into a sterile tube or a blood culture bottle.	[23]
			aspiration	sterile containers or syringes	-	-	[24]
ocular	conjunctiva		swab	pre-moistened swabs	-	roll swab across the conjunctival fornix	[18]
			spatula	flat, round-tipped spatula		swiftly scrape in one direction until fluid accumulates on the instrument edge	
			aspiration	fine needle		for conjunctival masses (round cell neoplasms, granulomas, abscesses)	
			swab	swab in transport medium	-	topical anaesthetic may inhibit bacterial growth	[23]
reproductive	vagina and uterus		biopsy or uterine washes	sterile container	-	vaginoscope, proctoscope or endoscopic biopsy port	[24]
	mammary gland		manual milk extraction	sterile container	-	clean and disinfect the gland and teat surface	
	testicles		second fraction of the ejaculate collection or cystocentesis	sterile containers	-	-	
	prostate		aspiration, biopsy, mid-third ejaculate fraction, rectal prostatic massage, or cystocentesis.	sterile containers	-	-	
	semen		swab	swab with transport medium	-	saturate swab; add 2–3 mL fluid to gel medium; submit excess in RTT	[10]
respiratory	upper airway	nostril	swab	moist cotton swab	-	-	[21]
			swab	cotton swab with Amies transport medium	-	-	[28]
		nasal cavity	swab	cotton swab with Amies transport medium	-	after cleaning and disinfection, swab advanced to the medial canthus of the eye and rotated	[28]
		mucosa	biopsy (either guided by endoscopy during rhinoscopy or blind)	sterilised biopsy forceps and transferred to a swab	-	flushed with sterile saline before sample collection	[28]
		sinus	swab or biopsy or aspiration	swab in transport medium or tissue/fluid in WTT	-	aspirate from maxillary, frontal or other sinuses	[23]
		multiple animal species	swab and biopsy via rhinoscopy	swabs and biopsies in transport medium *	-		[24]
	lower airway		BAL, tracheal wash	swab with transport medium	-	saturate swab; add 2–3 mL fluid to gel medium. Submit excess fluid in RTT.	[10]
			BAL	WTT or RTT	-	place wash fluid in a sterile tube.	[23]
			BAL or brush samples via bronchoscopy	transfer retrieved fluid to transport medium *	-	-	[24]
	pleural fluid		thoracocentesis and an indwelling thoracic catheter	Non-anticoagulant tube	-	aseptic technique	[25]
urinary	urine		cystocentesis	sterile, preservative-free, plastic tubes	-	-	[20]
				plain plastic container	5–6 mL	-	[22]
				WTT	5 mL	-	[23]
				sterile container or boric acid tubes	-	-	[24]
				-	-	ultrasound-guided	[26]
				-	3 mL	aseptically transferred to a WTT	[32]
				BD Vacutainer® Plus C&S Boric Acid Sodium Borate tubes (Becton Dickinson)	2 mL	ultrasound-guided	[33]
			urinary catheterization	plain plastic container	5–6 mL	-	[22]
				WTT	5 mL	-	[23]
				-	3 mL	aseptically transferred to a WTT	[32]
			midstream catch	sterile, preservative-free, plastic tubes	-	-	[20]
				plain plastic container	5–6 mL	-	[22]
				WTT	5 mL	-	[23]
				-	3 mL	aseptically transferred to a WTT	[32]

* Amies or Stuart medium, WTT: plain sterile container, RTT: Red-top tube (with clot activator).

**Table 3:** Antibiotic withdrawal periods before sample collection, according to the organ tract.

Reference	Antibiotic Withdrawal	Organ Tract
[10]	-	body cavities, circulatory, gastrointestinal, integumentary, musculoskeletal, reproductive, respiratory
[18]	collect samples before antibiotic therapy and topical anaesthetics	ocular
[19]	4 weeks	integumentary
[20]	-	urinary
[21]	-	respiratory
[22]	-	body cavities, musculoskeletal, urinary
[23]	minimum of 72 h (ideally 7–10 days)	central nervous system, circulatory, gastrointestinal, integumentary, musculoskeletal, ocular, respiratory, urinary
[24]	no withdrawal required; antibiotic therapy should be reported	body cavities, central nervous system, circulatory, gastrointestinal, integumentary, musculoskeletal, reproductive, respiratory, urinary
[25]	no withdrawal required; antibiotic exposure noted	respiratory
[26]	no withdrawal required; antibiotic exposure noted	urinary
[27]	72 h	integumentary
[28]	-	respiratory
[29]	no withdrawal required; antibiotic exposure noted	gastrointestinal
[30]	-	integumentary, gastrointestinal
[31]	-	gastrointestinal
[32]	-	urinary
[33]	4 weeks	circulatory, urinary

**Table 4:** Summary of sample storage conditions, submission time to the laboratory and bacterial groups targeted for culture in the selected studies.

Organ System	Sample Type		Bacterial Group Target	Storage	Time from Sample Collection to Laboratory Processing	References
Body cavities	effusions		aerobes (aerobic transport media or anaerobic transport media AND aerobic blood culture bottle) and/or anaerobes (anaerobic transport media AND anaerobic blood culture bottle)	(aerobe culture) refrigerated if aerobic transport medium or room temperature if aerobic blood culture bottle (anaerobe culture) room temperature	48 h or 72 h 72 h	[10]
			-	refrigerated frozen	<24 h >24 h	[22]
			aerobes and anaerobes	(aerobe culture) refrigerated (anaerobe culture) room temperature	24 h	[24]
Central Nervous	cerebrospinal fluid (CSF)		aerobes and/or anaerobes	room temperature	-	[23]
Circulatory	blood		aerobes or anaerobes	room temperature	72 h	[10]
			aerobes and/or anaerobes	room temperature	-	[23]
			aerobes and anaerobes	(aerobe culture) refrigerated (anaerobe culture) room temperature	24 h	[24]
			aerobes and anaerobes	refrigerated	<1 h	[33]
	bone marrow		aerobes and/or anaerobes	-	-	[23]
Gastrointestinal	oral mucosa		aerobes	refrigerated	-	[30]
	bile		aerobes and anaerobes	-	1 h	[29]
	faeces		aerobes and/or anaerobes and/or *Campylobacter jejuni*	(aerobe culture) refrigerated if plain container or aerobic transport medium; room temperature if anaerobic transport medium or modified Cary-Blair	24 h or 48 h 72 h or 96 h	[10]
				(anaerobes culture) room temperature	72 h	
				(*Campylobacter jejuni* culture) refrigerated if Amies with charcoal or room temperature if modified Cary-Blair	48 h or 96 h	
			aerobes and/or anaerobes	refrigerated or room temperature	-	[23]
			aerobes and anaerobes	(aerobe culture) refrigerated (anaerobe culture) room temperature	24 h	[24]
			microaerophilic (for *Campylobacter* spp. search)	refrigerated	-	[31]
	perianal		aerobes (for *S. pseudintermedius* search)	refrigerated (4 °C)	-	[30]
Integumentary	abscesses or wounds		aerobes and anaerobes	(aerobe culture) refrigerated (anaerobe culture) room temperature	48 h 72 h	[10]
			aerobes and/or anaerobes	-	-	[23]
			aerobes and anaerobes	(aerobe culture) refrigerated (anaerobe culture) room temperature	24 h	[24]
	crust		aerobes and anaerobes	(aerobe culture) refrigerated (anaerobe culture) room temperature	24 h	[24]
	epidermal collarette		aerobes and anaerobes	(aerobe culture) refrigerated (anaerobe culture) room temperature	24 h	[24]
	external ear canal		aerobes	-	-	[23]
			aerobes	refrigerated	24 h	[24]
			aerobes	refrigerated	-	[30]
	middle ear		aerobes	refrigerated	24 h	[24]
	nail		aerobes	-	-	[23]
	pustule		aerobes and anaerobes	(aerobe culture) refrigerated (anaerobe culture) room temperature	24 h	[24]
	pyoderma		aerobes and anaerobes	(aerobe culture) refrigerated (anaerobe culture) room temperature	24 h	[24]
	skin		aerobes and anaerobes	(aerobe culture) refrigerated (anaerobe culture) room temperature	48 h 72 h	[10]
			aerobes (for *Staphylococcus* spp. search)	-	-	[19]
			aerobes	-	-	[23]
			aerobes (for *S. pseudintermedius* search)	refrigerated (4 °C)	-	[30]
	surgical site		aerobes	processed immediately after collection	0 h	[27]
Musculoskeletal	tissue		aerobes and anaerobes	(aerobe culture) refrigerated (anaerobe culture) room temperature	48 h 72 h	[10]
			-	refrigerated frozen	<24 h >24 h	[22]
			aerobes and/or anaerobes	-	-	[23]
	post mortem tissue		aerobes and/or anaerobes and/or *Campylobacter jejuni*/*Salmonella*/*Yersinia*	refrigerated	-	[10]
	synovial fluid		aerobes and/or anaerobes	room temperature	<48 h	[23]
Ocular	conjunctiva		aerobes (anaerobes only for aspiration or biopsies)	refrigerated	-	[18]
			aerobes	-	-	[23]
Reproductive	vagina and uterus		aerobes	refrigerated	24 h	[24]
	mammary gland		aerobes	refrigerated	24 h	[24]
	testicles		aerobes (consider testing for brucellosis)	refrigerated	24 h	[24]
	prostate		aerobes	refrigerated	24 h	[24]
	semen		aerobes and/or anaerobes	(aerobe culture) refrigerated (anaerobe culture) room temperature	48 h 72 h	[10]
Respiratory	upper airway	nostril	aerobes	-	2–4 h	[21]
			aerobes	-	<24 h	[28]
		nasal cavity	aerobes	-	<24 h	[28]
		mucosa	aerobes	-	<24 h	[28]
		sinus	aerobes and/or anaerobes	-	-	[23]
		all	aerobes	refrigerated	24 h	[24]
	lower airway		aerobes and/or anaerobes	(aerobe culture) refrigerated (anaerobe culture) room temperature	48 h 72 h	[10]
			aerobes	-	-	[23]
			aerobes	refrigerated	24 h	[24]
	pleural fluid		aerobes and anaerobes	refrigerated (4 °C)	<8 h	[25]
Urinary	urine		aerobes	refrigerated	24 h (RTT) 48 h (WTT)	[10]
			aerobes	refrigerated (2–8 °C)	24 h	[20]
			-	room temperature (protected from UV light)	<30 min	[22]
			-	refrigerated (2–8 °C) (protected from UV/sunlight)	-	[23]
			aerobes	refrigerated room temperature	<24 h 4 h	[24]
			aerobes	refrigerated (4 °C)	<24 h	[26]
			aerobes	refrigerated (2–8 °C)	<24 h	[32]
			aerobes	refrigerated	<1 h	[33]

## Data Availability

No new data were created or analysed in this study. Data sharing is not applicable to this article.

## Appendix A

**Table A1:** Risk of bias assessment of cross-sectional, prevalence, and expert opinion studies (JBI tool).

Reference	Type of Study	Study Aim	Q1	Q2	Q3	Q4	Q5	Q6	Q7	Q8	RoB
[10]	Cross-sectional	Best practices for veterinary culture specimen handling.	Y	Y	Y	N/A	N/A	Y	N/A	Y	moderate
[18]	Expert opinion	Conjunctival cytology methods and findings in dogs and cats	N/A	Y	Y	Y	N/A	Y	N/A	N/A	moderate
[19]	Cross-sectional	Association of *Staphylococcus* spp. with superficial pyoderma and antimicrobial resistance in allergic cats	Y	Y	Y	Y	U	N	Y	Y	moderate
[21]	Cross-sectional	Prevalence and antimicrobial susceptibility of commensal *Staphylococcus* spp.	Y	Y	Y	Y	U	Y	Y	Y	low
[25]	Case report	Clinical and microbiologic comparison in cats and dogs with pyothorax.	Y	Y	Y	Y	U	Y	Y	Y	low
[26]	Cross-sectional	Prevalence and antimicrobial susceptibility of uropathogens in dogs and cats.	Y	Y	Y	Y	Y	Y	Y	Y	low
[27]	Cross-sectional	Prevalence and resistance of bacteria in the surgical environment.	Y	Y	Y	Y	U	U	Y	Y	moderate
[30]	Cross-sectional	Prevalence, resistance, and virulence genes of *S. pseudintermedius* in cats.	Y	Y	Y	Y	U	Y	Y	Y	low
[31]	Cross-sectional	Isolation and antibiograms of *Campylobacter jejuni* and *C. coli* in pets.	Y	Y	Y	Y	U	U	Y	Y	moderate
[33]	Prevalence data	Occurrence of bacteraemia and bacteriuria in dogs and cats with CKD.	Y	Y	Y	Y	U	Y	Y	U	moderate

Q1, Clear inclusion criteria, Q2, Detailed description of setting and participants, Q3, Valid and reliable sampling method, Q4, Standardised and reliable measurement, Q5, Handling of confounding factors, Q6, clear reporting of sample handling/transport, Q7, Outcome measured consistently and objectively, Q8, Adequate statistical analysis, RoB, Risk of bias, Y, yes, N, no, U, uncertain, N/A, not applicable.

**Table A2:** Risk of bias assessment of diagnostic accuracy studies (QUADAS-2 tool).

Reference	Type of Study	Study Aim	Q1	Q2	Q3	Q4	Q5	RoB
[20]	Diagnostic accuracy	Compare urine culture and susceptibility results	Y	Y	Y	Y	Y	low
[28]	Diagnostic accuracy	Assess the agreement of bacterial culture from nasal discharge, cavity, and biopsy	Y	Y	Y	Y	Y	low
[29]	Diagnostic accuracy	Evaluate the agreement between microscopic examination and bile culture for bactibilia detection.	Y	Y	Y	Y	Y	low
[32]	Diagnostic accuracy	Assess the effect of urine specific gravity on predicting culture results via microscopy	Y	Y	Y	Y	Y	low

Q1, Patient selection, Q2, Index test, Q3, Reference standard, Q4, Flow and timing, Q5, Applicability concerns, RoB, Risk of bias, Y, yes.

**Table A3:** Risk of bias assessment of clinical practice guidelines (AGREE II tool).

Reference	Type of Study	Study Aim	Q1	Q2	Q3	Q4	Q5	Q6	RoB
[22]	Clinical practice guideline	best clinical practices	Y	U	U	Y	Y	U	moderate
[23]	Clinical practice guideline	best clinical practices	Y	U	N	Y	Y	U	moderate
[24]	Clinical practice guideline	best clinical practices	Y	Y	Y	Y	Y	Y	low

Q1, Scope and purpose, Q2, Stakeholder involvement, Q3, Methodology rigour, Q4, Clarity of presentation, Q5, Applicability, Q6, Editorial independence, RoB, Risk of bias, Y, yes, N, no, U, uncertain.

**Table A4:** Absolute frequencies of bacterial groups targeted for culture (aerobes, anaerobes, and *Campylobacter* spp.), according to storage time and temperature conditions (refrigerated and room temperature).

	Aerobes					Anaerobes					*Campylobacter* spp.				
Time	R	RT	F	NM	T	R	RT	F	NM	T	R	RT	F	NM	T
<8 h	3	1	0	5	9	1	0	0	0	1	0	0	0	0	0
24 h	22	0	0	0	22	0	8	0	0	8	0	0	0	0	0
48 h	8	1	0	0	9	0	1	0	0	1	1	0	0	0	1
72 h	0	3	0	0	3	0	8	0	0	8	0	0	0	0	0
96 h	0	1	0	0	1	0	0	0	0	0	0	1	0	0	1
NM	7	3	0	0	10	3	3	0	0	6	2	0	0	0	2
T	40	9	0	5	54	4	20	0	0	24	3	1	0	0	4

Values represent the number of samples (n), R: refrigerated, RT: room temperature, F: frozen, NM: not mentioned, T: total.
